# Contused Wounds of the Abdomen

**Published:** 1898-11-05

**Authors:** 


### CONTUSED WOUNDS OF THE ABDOMEN.

In a paper read at the annual meeting of the Minnesota State Society by Dr. John T. Rogers, and reported in the New York *Medical Record*, the treatment of severe injuries to the abdomen is carefully considered. A symptom of prime importance in these cases is shock, which is almost always present. In fact, a blow over the solar plexus may cause death by shock, even where there is no external evidence of violence to the abdominal wall. Severe pain, nausea and vomiting, immediately following or accompanying shock, may be taken as evidence of grave abdominal injury, although cases are reported in which none of these symptoms were present. Usually, however, there is intense pain and the vomiting is continuous. Extreme rigidity of the abdominal walls is, perhaps, the most important and characteristic symptom. It is present in every case, and may be considered almost pathognomonic of grave intra-abdominal lesion. In his interesting paper read before the Medical Society of London last week, Mr. Marmaduke Sheild referred to the same thing, pointing out the importance of abdominal rigidity as a symptom in this class of injury. In the presence, then, of such symptoms what are we to do? Dr. Rogers says, "Without immediate surgical interference I believe that not one in one hundred cases of severe abdominal contusion ends in recovery. . . . . Without operation these patients, when they recover from the primary shock, rapidly develop septic general peritonitis and die, or else, especially in injuries to the liver and spleen, they die of hæmorrhage;" and he goes on to say: "My experience with abdominal contusions, and a careful study of all the literature at my disposal, teach me to say unhesitatingly that the most conservative treatment is early operation, and I mean by 'early operation' as soon after the injury as possible; immediately if the patient is not in collapse. . . . . . I believe it is better surgery to open the abdomen in doubtful cases and find no lesion, than to wait in a single instance until signs of septic peritonitis are present. An aseptic operation will do no harm, while delay means death in 96 per cent. of all cases of rupture of the intestine." To locate the injury is often by no means an easy matter, and in endeavouring to do so it is well to bear in mind the exact point at which the violence was applied, for, in consequence of the paralysis which supervenes on such an injury, the rupture will in most cases "be found immediately beneath the point where the vulnerating force came in contact with the abdominal wall." When the rupture is found, Dr. Rogers is in favour of pulling out only so much of the intestine as is required for the closure of the rent, and of the removal of all septic material by carefully mopping out the cavity with soft gauze sponges. "Practical experience in irrigating in cases of septic peritonitis convinces me," he says, "that it should be abandoned. Gently mopping out the cavity and thorough gauze drainage will give better results." For the control of hæmorrhage, in cases where the liver or spleen is lacerated, packing is the best plan. Great care should be exercised to keep the patient warm by hot bottles during all such operations, and infusion with saline fluid is almost always indicated both during and after operation.

